# PENELOPE/PRIMO-calculated photon and electron spectra from clinical accelerators

**DOI:** 10.1186/s13014-018-1186-8

**Published:** 2019-01-11

**Authors:** Lorenzo Brualla, Miguel Rodriguez, Josep Sempau, Pedro Andreo

**Affiliations:** 1 West German Proton Therapy Centre Essen (WPE), Essen, D-45147 Germany; 2 West German Cancer Center (WTZ), Essen, D-45147 Germany; 3 University Hospital Essen, Essen, D-45147 Germany; 4 Universität Duisburg-Essen, Medizinische Fakultät, Essen, D-45147 Germany; 5 Centro Médico Paitilla, Panama City, 0816-03075 Panama; 6 Instituto de Investigaciones Científicas y de Alta Tecnología, INDICASAT-AIP, City of Knowledge, Building 219, Panama City, Panama; 7 Department of Physics and Institute of Energy Technologies, Universitat Politècnica de Catalunya, Barcelona, E-08028 Spain; 8 Department of Medical Radiation Physics and Nuclear Medicine, Karolinska University Hospital, and Department of Oncology-Pathology, Karolinska Institutet, Stockholm, SE-171 76 Sweden

**Keywords:** Monte Carlo, PENELOPE, penEasy, PRIMO, Linac spectra, Radiotherapy physics, Radiotherapy dosimetry, Radiotherapy accelerators

## Abstract

**Background:**

The availability of photon and electron spectra in digital form from current accelerators and Monte Carlo (MC) systems is scarce, and one of the packages widely used refers to linacs with a reduced clinical use nowadays. Such spectra are mainly intended for the MC calculation of detector-related quantities in conventional broad beams, where the use of detailed phase-space files (PSFs) is less critical than for MC-based treatment planning applications, but unlike PSFs, spectra can easily be transferred to other computer systems and users.

**Methods:**

A set of spectra for a range of Varian linacs has been calculated using the PENELOPE/PRIMO MC system. They have been extracted from PSFs tallied for field sizes of 10 cm × 10 cm and 15 cm × 15 cm for photon and electron beams, respectively. The influence of the spectral bin width and of the beam central axis region used to extract the spectra have been analyzed.

**Results:**

Spectra have been compared to those by other authors showing good agreement with those obtained using the, now superseded, EGS4/BEAM MC code, but significant differences with the most widely used photon data set. Other spectra, particularly for electron beams, have not been published previously for the machines simulated in this work. The influence of the bin width on the spectrum mean energy for 6 and 10 MV beams has been found to be negligible. The size of the region used to extract the spectra yields differences of up to 40% for the mean energies in 10 MV beams, but the maximum difference for TPR _20,10_ values derived from depth-dose distributions does not exceed 2% relative to those obtained using the PSFs. This corresponds to *k*_*Q*_ differences below 0.2% for a typical Farmer-type chamber, considered to be negligible for reference dosimetry. Different configurations for using electron spectra have been compared for 6 MeV beams, concluding that the geometry used for tallying the PSFs used to extract the spectra must be accounted for in subsequent calculations using the spectra as a source.

**Conclusions:**

An up-to-date set of consistent spectra for Varian accelerators suitable for the calculation of detector-related quantities in conventional broad beams has been developed and made available in digital form.

**Electronic supplementary material:**

The online version of this article (10.1186/s13014-018-1186-8) contains supplementary material, which is available to authorized users.

## Introduction

It is well-known that a comprehensive phase-space file (PSF) characterizing the energy, position, direction and statistical weight of all the particle generations emerging from a clinical accelerator and reaching the surface of a phantom or a patient, provides a suitable source for Monte Carlo (MC) radiotherapy dosimetry calculations. These include the calculation of detector-related quantities in and patient treatment planning (see references [1, 2]). PSFs should include a very large number of particles to minimize as much as possible the so-called latent variance [3] in the calculated quantity, although strictly the statistical variance is only part of the simulated story, since it should be combined with the type A uncertainty of the MC calculation. Hence, PSFs are usually very large, of the order of gigabytes (GB), their size being an inconvenient at the time of a data transfer or exchange among users.

PSFs are necessary for MC simulations involving the extended 3-D geometries of patient CT data, but the necessity can be relaxed to some extent for the simulation of detector-related quantities, as those included in dosimetry protocols [4–8], since equivalent results are obtained [9]. For the latter, using the “main incident particle” spectrum, i.e., photons or electrons, has become the most common practice. It should be noticed that spectra extracted from a small central region of a broad beam is not equivalent to that obtained from the simulation of a small beam.

The most widely used set of clinical accelerator photon spectra for conventional broad beams is that calculated by Mohan et al. [10], henceforth referred to as Mohan, for four Varian Clinac machines with acceleration potentials between 4 MV and 24 MV using the EGS4 MC system [11]. A reason for its frequent use is that the set of spectra is included in digital form in the EGSnrc [12] distribution package since long ago. A set of nine photon broad beam spectra from accelerators manufactured by Elekta, Siemens and Varian was published by Sheikh-Bagheri and Rogers [13], henceforth referred to as SBR; they were also calculated with the EGS4 MC system, using its BEAM code [14]. These spectra, unlike those of Mohan, were presented in tabular form with a bin width of 250 keV; however, they were not included in the EGSnrc package and, probably, due to this reason their use has been rather limited.

Electron broad beam spectra have, on the other hand, received much lower attention, to the extent that no detailed tabulation has been made available in the peer-reviewed literature. An internal report by Ding and Rogers [15], henceforth referred to as DR, included a large set of electron spectra in the energy range of 5–50 MeV from various accelerators. They were also calculated with the EGS4/BEAM code and presented in graphical form, but despite its introductory statement on digital availability, neither the report nor the data can be found in the Internet address provided or in the EGSnrc or the National Research Council of Canada web sites. The set is not included either in the current EGSnrc package.

The IAEA has also developed a database of PSFs [16] that includes ^60^Co *γ* rays and a number of accelerator photon and electron beams (https://www-nds.iaea.org/phsp) from where spectra can be extracted, but the range of beam energies available is rather limited. The database has not been updated since 2013.

Considering that many of the photon and electron spectra mentioned are from accelerators no longer in clinical use, and the general lack of availability of their data in digital form, a project was undertaken to produce a consistent set of the two types of spectra for conventional broad beams from current accelerators. The spectra are extracted from PSFs calculated with the MC system PENELOPE/PRIMO [17–19]. This work provides a description of the methods and calculations performed to derive the data and provides tables of spectra for 6–22 MeV electron beams and 6–20 MV photon beams, the latter group including two flattening-filter-free (FFF) beams in frequent clinical use. The work also includes the analysis of the influence of the spectral bin size and the size of the region around the beam central axis used to extract the spectra for 6 and 10 MV. Comparisons with other published spectra are made whenever data for the same or similar linacs are available. The spectra in digital form are available in the PRIMO web site (https://www.primoproject.net).

## Material and methods

The calculations presented in this work have been performed with the PRIMO software (version 0.3.1.1681) [18, 19], which is based on the PENELOPE (version 2011) / penEasy Monte Carlo code.

PENELOPE [20] is a general-purpose MC system for the simulation of the transport of photons and electrons in arbitrary media. Its early use for simulating linac electron beams goes back to 2001 [3]. PENELOPE provides the calculation engine for a number of codes developed for the simulation of the treatment head of different accelerators, such as PENLINAC [21], PENEASYLINAC [22] and PRIMO. PENELOPE is in fact a routine library which requires a steering main program to provide, among other aspects, the description of the particle source, the tallies to be scored and certain variance-reduction techniques (VRT) that are applied at the main program level. The penEasy code [22] is one of such main programs for PENELOPE, which includes the handling of voxelized geometries. PRIMO is a free-software package that simulates clinical linacs and estimates absorbed dose distributions in phantoms and computerized tomographies; it combines a graphical user interface with the PENELOPE/penEasy system.

The spectra presented in this work have been extracted from PSFs tallied with PRIMO. Flattening filtered photon beams of 6, 10, 15 and 20 MV from a Varian Clinac C series (e.g., models 2100, 18, 1800, 2300 and iX) were simulated with a field size of 10 cm × 10 cm. FFF photon beams of 6 and 10 MV from a Varian TrueBeam were simulated using the FakeBeam empirical geometry described by Rodriguez et al. [23], also with a field size of 10 cm × 10 cm. Electron beams of 6, 12, 18 and 22 MeV from the same Clinac C series were simulated with a field size of 15 cm × 15 cm collimated with the electron applicator. The extracted spectra, averaged over either the entire beam area or restricted to a narrow region around the beam central axis, were tallied in 250 keV-, 50 keV- and 1 keV-wide bins, depending on the case.

The number of incident particles simulated was 10^9^ and 10^10^ for photon and electron beams, respectively, leading to PSFs of the order of 10^8^– 10^9^ particles. The size of the PSFs were 60–360 GB for the electron beams and 4–64 GB for photons. The speed and accuracy of the particle MC simulation was controlled using the following transport parameters:

(i) Particle cut-off energies, below which the transport of particles is halted, was set to 200 keV for electrons and positrons, and to 50 keV for photons.
(ii) The limits between detailed and condensed simulation of charged particles are governed in PENELOPE by *W*_CC_, *W*_CR_, *C*_1_ and *C*_2_. The first two parameters set the limit of the energy loss thresholds separating hard and soft events for electronic collisions and bremsstrahlung emission, respectively. *C*_1_ and *C*_2_ are related with the corresponding angular threshold for elastic deflections (refer to the PENELOPE manual for further details). For electron beams their values were *W*_CC_=200 keV, *W*_CR_=50 keV and *C*_1_=*C*_2_=0.1. For photon beams the same transport parameters were used with the exception of *W*_CR_=200 keV for all materials and *C*_1_=*C*_2_=0.001 in the bremsstrahlung accelerator target.
(iii) The parameter DSMAX, defining the maximum step length allowed for electrons and positrons, was set to 1/10 of the thickness of each component of the linac.
(iv) All the primary particle sources (for both electron and photon beams) were modeled as monoenergetic point sources with zero divergence.
(v) The accelerator incident electron energies were those set as default in PRIMO, which are a good ansatz for most Varian Clinac C series machines. For electron beams, the energies used were 6.85 MeV (for 6 MeV), 13.37 MeV (for 12 MeV), 19.97 MeV (for 18 MeV) and 24.46 MeV (for 22 MeV). For photon beams they were 5.4 MeV (for 6 MV), 10.5 MeV (for 10 MV), 14.3 MeV (for 15 MV) and 18.5 MeV (for 20 MV).
(vi) The variance reduction techniques used to speed up the photon simulations were splitting roulette [24] for 6 and 10 MV, and rotational splitting [25] for 15 and 20 MV. No VRTs were used for the simulation of electron beams.
(vii) Electron PSFs were tallied downstream of the third scrapper of the electron applicator defining the 15 cm × 15 cm field size at the phantom surface. Photon PSFs were tallied at the exit of the gantry for a 10 cm × 10 cm field size at the phantom surface. The field size was defined at isocenter distance, as usual.

## Results and discussion

**Fig. 1:**
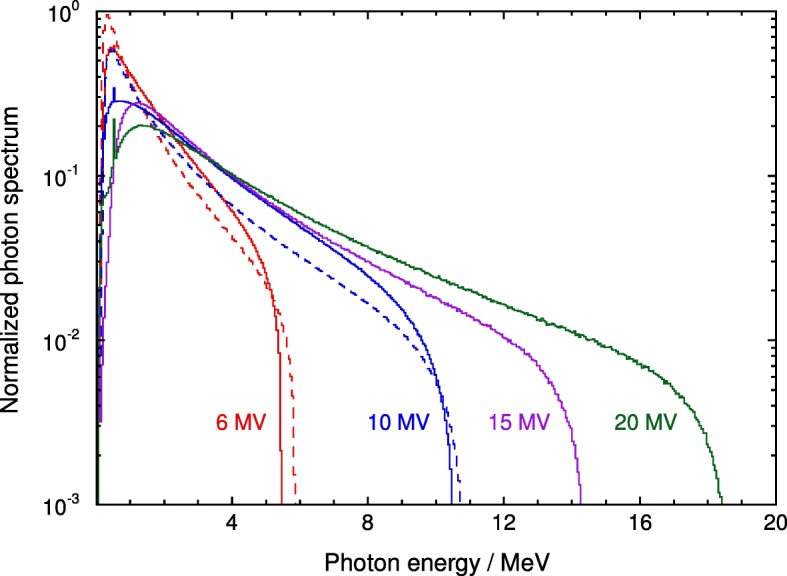
Calculated photon spectra (planar fluence differential in energy) for 6, 10, 15 and 20 MV Varian clinical beams normalized to their integral. The dashed histograms correspond to spectra for 6 and 10 MV FFF beams. Observe the 511 keV peak resulting from positron annihilation, visible in the higher energy beams

**Fig. 2:**
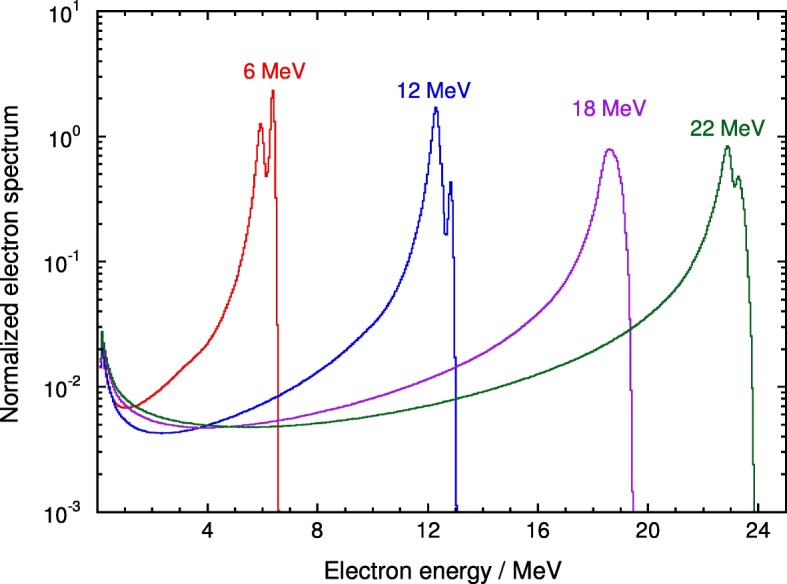
Calculated electron spectra (planar fluence differential in energy) for 6, 12, 18 and 22 MeV Varian clinical beams normalized to their integral

The PSF-extracted spectra (strictly, planar fluence differential in energy [8]) for the photon and electron beams are shown in Figs. 1 and 2, respectively, where, to enable comparison, they have been normalized to their respective integrals. To facilitate the visualization of the spectra, uncertainties are not shown in the plot but the smoothness of the data indicates a rather low statistical uncertainty (type A). The spectra are given in numerical form in the Additional files 1 and 2, which includes the standard uncertainty of the fluence in each bin as a percentage of the value.

The bin width of the spectra shown in Figs. 1 and 2 is 50 keV. This width allows the visualization of 511 keV photons resulting from positron annihilation, which are visible in the higher energy beams of Fig. 1, but would be almost unnoticeable using a broader width (see, e.g., figure 10 in SBR [13], where a bin width of 250 keV was used).

**Fig. 3:**
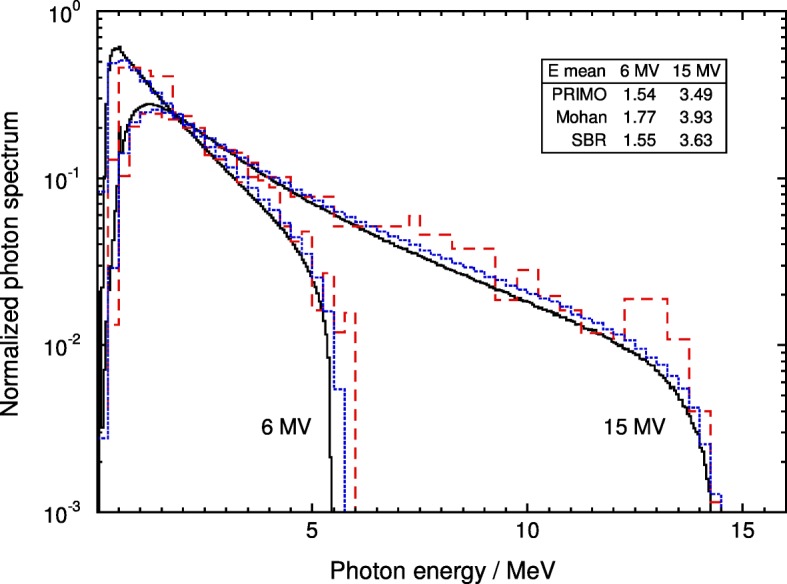
Comparison of the PENELOPE/PRIMO photon spectra for Varian beams of 6 and 15 MV with those calculated by Mohan et al. [10], dashed histograms, and Sheikh-Bagheri and Rogers [13], dotted histograms. All the spectra are normalized to their respective integral. The fluence-weighted mean energies of each spectrum are indicated in the inset

Our calculated photon spectra are compared with those of Mohan [10] and SBR [13] for Varian broad beams of 6 and 15 MV in Fig. 3, where the mean energies of the different spectra are indicated. These authors derived the spectra from a broad beam while recording the particles in a small central region of radii 3 cm and 2.25 cm, respectively, whereas in the present work the spectra are for a 10 cm × 10 cm field in which in the indicated cases particles from the whole field are recorded; this alternative should make their beams slightly harder than ours (see, e.g., refs. [5, 26]). There are large differences in shape with Mohan’s spectra. Additionally, their broad bin widths show a rather large uncertainty, and the mean energies differ from those in the present work by 15% (6 MV) and 12.6% (15 MV). The PRIMO spectra do not differ substantially from those of SBR [13] although slightly higher mean energies can be observed in consistency with the comment above regarding small fields; despite their broader bin width (250 keV versus our 50 keV) the mean energies differ by only 0.4% for the 6 MV beam, while the difference is 4.1% for 15 MV. Not shown in the plot to avoid cluttering, for 10 MV the differences in mean energies are 7.5% for Mohan and 6.7% for SBR. It should also be noted that the differences mentioned, particularly in Mohan’s case, could be related to changes in the linac modeling [27].

**Fig. 4:**
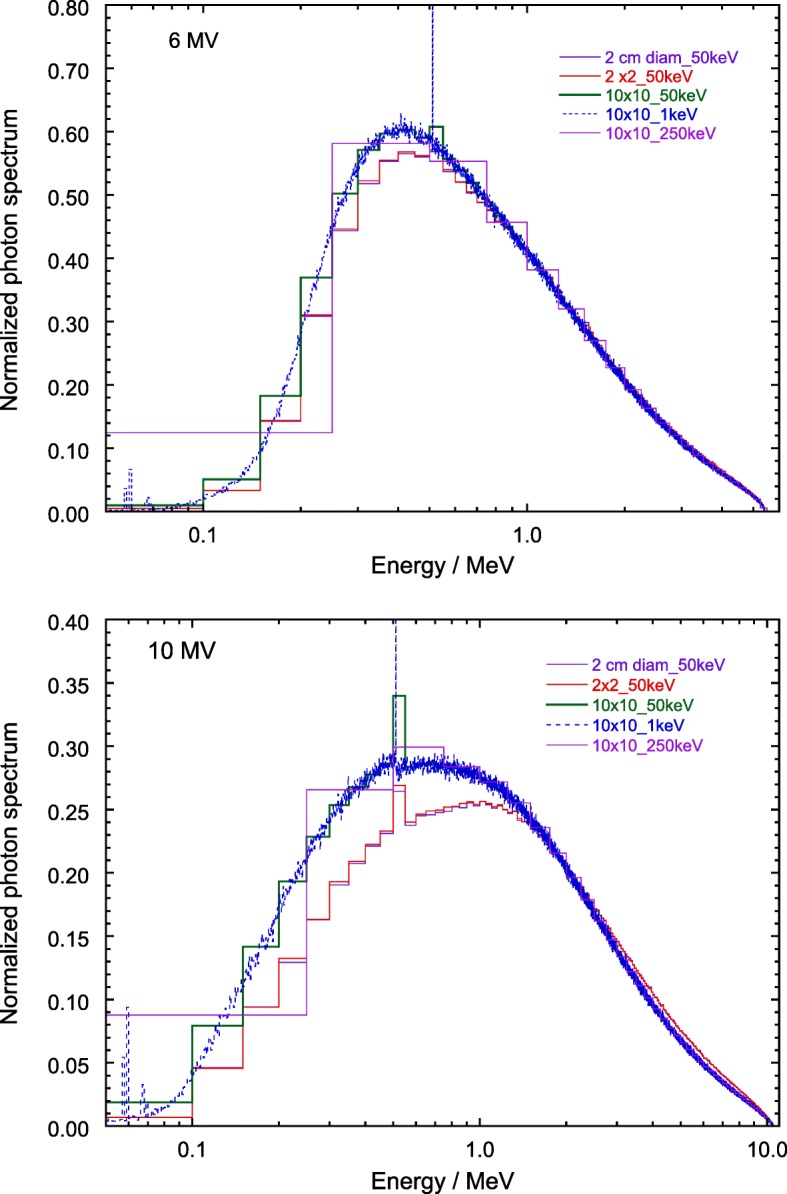
Photon spectra for 6 and 10 MV beams, derived from the PSF for a 10 cm × 10 cm field, for a central circular area of 2 cm diameter and a square of 2 cm side using bin widths of 50 keV, and for a square of 10 cm side using bin widths of 1, 50 and 250 keV. All the spectra are normalized to their respective integral. Note the energy logarithmic scale for better visualization

The influence of the region size around the central beam axis used to extract the photon spectrum from a 10 cm × 10 cm PSF, and of the bin width of the spectrum have been investigated for 6 and 10 MV photon spectra obtained for different conditions. These have been a circular area of 2 cm diameter and a square of 2 cm side for bin widths of 50 keV, and a square of 10 cm side for bin widths of 1, 50 and 250 keV. The set of five spectra are shown in Fig. 4 for both beam nominal energies, and the corresponding fluence-weighted and energy fluence-weighted mean energies are given in Table 1.

**Table 1:** Fluence-weighted $\left (\bar E_{\Phi }\right)$ and energy fluence-weighted $\left (\bar E_{\Psi }\right)$ mean energies of 6 and 10 MV photon spectra for a central circular area of 2 cm diameter and a square of 2 cm side using bin widths of 50 keV, and for a square of 10 cm side using bin widths of 1, 50 and 250 keV

	Bin width	6 MV		10 MV	
Field size	(keV)	$\bar E_{\Phi }$ (MeV)	$\bar E_{\Psi }$ (MeV)	$\bar E_{\Phi }$ (MeV)	$\bar E_{\Psi }$ (MeV)
$\varnothing $ 2 cm	50	1.604	2.456	3.000	4.664
$\square $ 2 cm side	50	1.601	2.453	2.989	4.655
$\square $ 10 cm side	50	1.542	2.398	2.760	4.471
$\square $ 10 cm side	1	1.542	2.398	2.218	3.238
$\square $ 10 cm side	250	1.542	2.401	2.760	4.473

All the spectra are extracted from the PSF files for a 10 cm × 10 cm field

It can be observed in the table that for the 6 MV beams the dependence of the mean energy on the size of the extracting region is about 4% and 2% for $\bar E_{\Phi }$ and $\bar E_{\Psi }$, respectively, while the dependence on bin width is practically negligible. This is not the case, however, for the 10 MV beam, where differences respect to the size of the extracting region are of about 9% and 4% for $\bar E_{\Phi }$ and $\bar E_{\Psi }$, respectively, whereas for the bin width they are approximately 25% and 38%, respectively. The influence of the size of the extracting region could be inferred from the spectra in Fig. 4, which for the 10 MV beam shows a clear shift of the most probable energy towards higher values for small extraction regions. This is consistent with the 3-D spatial energy distributions of the 6 and 10 MV PSFs, as that for 10 MV shows a curvature that corresponds to higher energies at the beam center than in the periphery. For this reason, the spectra tabulated in the Additional file 1: Appendix are extracted from the respective PSFs for the entire field size, 10 cm × 10 cm and 15 cm × 15 cm for photons and electrons, respectively.

**Fig. 5:**
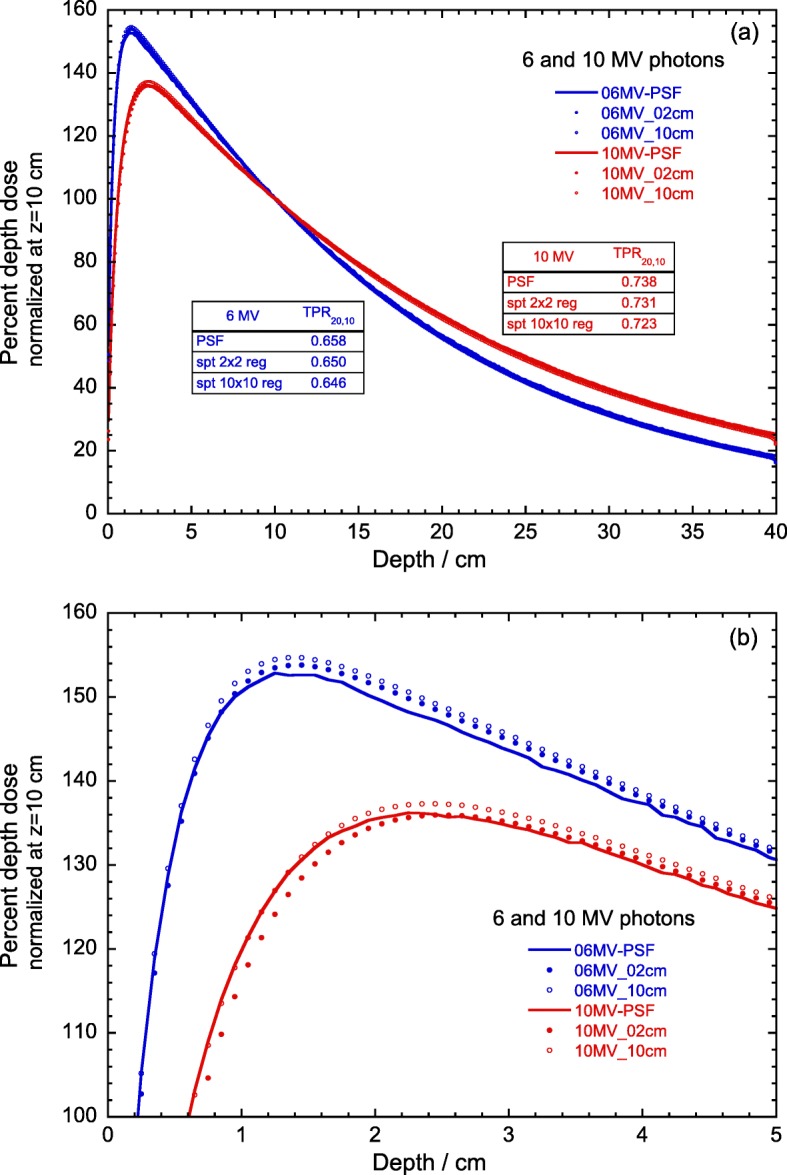
(**a**) Comparison of 6 and 10 MV photons depth-dose distributions, normalized at the depth of 10 cm, for 10 cm × 10 cm fields obtained from the PSFs (solid lines) with those obtained using the spectrum in a 2 cm × 2 cm central region around the beam central axis (filled circles) and in 10 cm × 10 cm (open circles). Panel (**b**) is an enlargement of the region around the maximum of the distributions. In all cases the quantity scored in the Monte Carlo simulations is the energy deposition in a region of 1 cm × 1 cm around the beam axis. The corresponding values of TPR _20,10_ are given in the insets of panel (**a**)

In spite of the differences mentioned, the mean photon energy of a MV spectrum is not a parameter used in reference dosimetry, as beams with the same mean energy might have different penetration properties (as it occurs, for example, with kV x-ray beams). Hence, it is of interest to verify how the beam quality index TPR _20,10_ for a 10 cm × 10 cm field, calculated with the spectra in the central region (e.g., 2 cm × 2 cm around the beam central axis) and in the entire field size, compare with the value obtained using the PSF. The rationale for this comparison is that spectra used for the simulation of divergent beams, where a point source emitting the spectrum irradiates the solid angle subtended by the field size, neglect the correlation between energy, position and direction of the incident photons. The correlation is thus ignored in both spectra but is taken into account when the PSF is used, which in addition includes contaminant electrons and positrons. Depth-dose distributions for this analysis are shown in Fig. 5 for 6 and 10 MV, where the respective TPR _20,10_ values are indicated in the insets. These have been obtained from exponential fits between 5 cm and 25 cm depth, yielding PDD _20,10_, and using the empirical relation between TPR _20,10_ and PDD _20,10_ given in IAEA TRS-398 [4]. It was found that, for the small region spectra, the TPR _20,10_ values differ by 0.8% and 0.9% for 6 and 10 MV, respectively. For the entire field spectra, the differences were 1.2% and 1.9%, respectively. However, considering for example a Farmer-type NE-2571 ionization chamber, these TPR _20,10_ correspond to *k*_*Q*_ differences between -0.06% and -0.25% relative to those for the PSF, which being considerably smaller than the standard uncertainty of *k*_*Q*_ (1%) can be considered to be negligible for MV reference dosimetry. Figure 5b shows the minor effect of neglecting the correlation between energy, position and direction of the incident photon spectra, where a small difference in the height of the maximum doses and their depths can be noted.

**Fig. 6:**
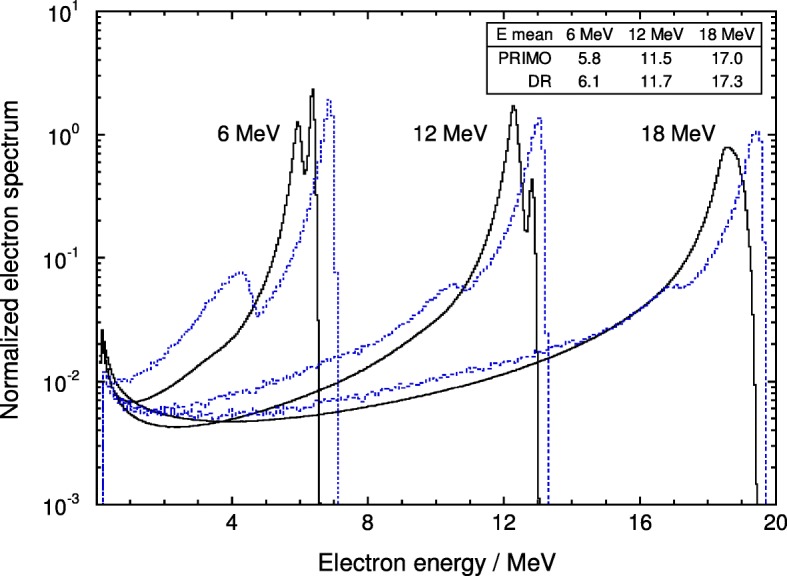
Comparison of the PENELOPE/PRIMO electron spectra for Varian beams of 6, 12 and 18 MeV with those calculated by Ding and Rogers [15], dotted histograms. All the spectra are normalized to their respective integral. The fluence-weighted mean energies of each spectrum are indicated in the inset. Note that the two linacs were not identical (see text)

For electron beam spectra a detailed comparison is not feasible due to the lack of data. However, spectra from the internal report by DR [15] mentioned in the introduction have been obtained. They had been extracted from PSFs calculated with EGS4/BEAM and are compared to those in the present work in Fig. 6.

It should be stressed, however, that the DR data do not correspond to a conventional Varian linac, as the particular Clinac 2100 C simulated in their study had thicker scattering foils and monitor chamber walls in order to match the depth-dose distributions of an earlier clinical machine (in Wisconsin). Further, for the energies compared in Fig. 6, their field size was 10 cm × 10 cm, while 15 cm × 15 cm was used in the present work. It can be observed in the figure that the two spectra datasets differ substantially but, from the comments above, significant differences were anticipated as the two linacs were not identical.

**Fig. 7:**
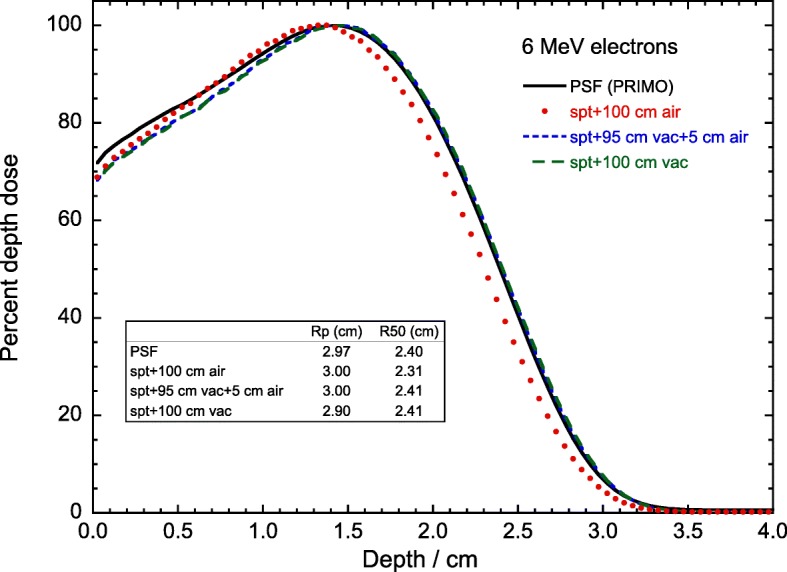
Comparison of 6 MeV electrons depth-dose distribution for a 15 cm × 15 cm field, obtained from the PSF (solid line) with those obtained using the spectrum plus an added 100 cm air filtration (dots), 100 cm of vacuum filling the space between the source and the phantom (long dashes), and 95 cm vacuum followed by 5 cm of air (short dashes). The corresponding values of *R*_50_ and *R*_p_ are given in the inset

The conditions under which a spectrum is used in a MC calculation have also been analyzed for a 6 MeV electron beam. As PSFs are tallied at the downstream end of the electron applicator, the simulation includes the air between the phantom surface and the electron applicator. Hence, a spectrum derived from the PSF includes the effect of air filtration. Sometimes, however, this condition is not properly accounted for and a point source is simulated at a certain SSD with air filling the space between the source and the phantom. This produces a double counting of the air effect, whose filtration can be of significance. Figure 7 compares the full PSF depth-dose distribution with those obtained using the spectrum under various configurations. The effect of an added 100 cm air filtration produces a less penetrating beam, which yields an incorrect depth-dose distribution. Using vacuum filling the space between the source and the phantom, or 95 cm vacuum followed by 5 cm of air (which is closer to the geometry for tallying the PSF), differs only by a few tenths of a percent from the PSF beam quality index *R*_50_ and practical range *R*_p_, the results for both geometries being indistinguishable. The region in front of the depth-dose maximum differs, however, from that for the PSF because the correlation between energy, position and direction of every particle, as well as contaminating particles, are ignored in an incident electron spectrum.

## Conclusions

An up-to-date set of consistent photon and electron spectra for a range of Varian accelerators has been calculated using the PENELOPE/PRIMO MC system. They have been extracted from PSFs calculated for field sizes of 10 cm × 10 cm and 15 cm × 15 cm for photon and electron beams, respectively, at an SSD of 100 cm using a bin width of 50 keV. Their use is intended for the simulation of detector-related quantities in conventional broad beams, where the use of detailed PSFs is less critical than for Monte Carlo-based treatment planning applications, but unlike PSFs, spectra can easily be transferred to other computer systems and users. They are provided in detailed tables and made available in digital form at the PRIMO web site for easy retrieval.

Spectra from this work have been compared to those obtained by other authors, showing rather good agreement with those calculated with the, now superseded, EGS4/BEAM MC system, but significant differences with the widely used “classic” photon data set from Mohan et al. (1985), available in the EGSnrc distribution package. Other spectra sets, particularly for electron beams, have not been previously published for the machines simulated in this work.

The influence of the bin width of the spectra extracted from the PSF has been investigated for 6 and 10 MV photon spectra using 1, 50 and 250 keV widths. Their impact on the fluence-weighted and energy fluence-weighted mean energies, $\bar E_{\Phi }$ and $\bar E_{\Psi }$, respectively, has been found to be negligible. The effect of the size of the region around the beam central axis used to extract the spectra, a narrow zone or the entire beam size, has been analyzed for these beams. For 6 MV, differences of about 4% and 2% for $\bar E_{\Phi }$ and $\bar E_{\Psi }$, respectively, have been found; the differences become 25% and 38% for the 10 MV beam. A comparison between depth-dose distributions for a 10 cm × 10 cm field calculated with these spectra and those obtained from the PSFs yields differences in TPR _20,10_ values between 0.7% and 1.9%. However, these correspond to negligible differences (up to 0.25%) in the *k*_*Q*_ values for a Farmer-type NE-2571 ionization chamber. The effect of disregarding the correlation between energy, position and direction in the incident photon spectra is rather small. It could be of some importance for distributions normalized at the depth of the maximum dose (depth doses or tissue-maximum ratios) due to the minor difference in the height and depth of the maxima, but is irrelevant for TPR distributions.

Different configurations for using electron spectra have been compared for 6 MeV beams, concluding that the geometry used for tallying the PSFs used to extract the spectra must be accounted for in subsequent calculations using the spectra as a source. The jeopardy of over counting the influence of air filtration has been emphasized.

## Additional files

Additional file 1 Table spectra.tex. The table is provided as a LaTeX file. Preferably it should be published as an Appendix; otherwise please make it available as an additional file in pdf. (TEX 60 kb)

Additional file 2 Table spectra.xlsx. The table is provided as an Excel “.xlsx” file. It should be made available as an additional file in Excel format. (XLSX 123 kb)

## Abbreviations

FFF: Flattening-filter-free
MC: Monte Carlo
PSF: Phase-space file
VRT: Variance-reduction technique
